# Diseases Admitted as in and Out-Patients of the Physicians of the Westminster Hospital, from the 20th of March to the 20th of April, 1799

**Published:** 1799-05

**Authors:** 


					General Remarks on the prevailing Difeafes of London, 233
jD IJ,cafes admitted, as in and out-Patients of the Phyficians of the JVcflmin-
Jier Hofpital, from the loth of March to the 20th of dpril, 1799*
No. of Cases.
Fevers 4
Amenorrhoea 8
Anafarca 2
Afcites 3
Afthenia 1
Afthma
Catarrh 1
Cholera 2
Convulfions I
Cephala:a 1
Cough - - "H
Diarrhoea 8
Dyfentery 3
Dyfpepfia 4
Dyfuria I
Enteroclynia -
Epilepfy
Eryfipelas
Hooping-cough
Hyiteria
Itch
Menorrhagia
Phthifis
Paralyfis
Pleurify
Quinfey
Rhcumatifm -
Struma -
Urticaria
Vomiting
No. of Cases.
2
1
l
I
1
- 6
i
- 4
- 4
- 5
2
" I?
- I
I
- i - I

				

